# GS-DTI: a graph-structure-aware framework leveraging large language models for drug–target interaction prediction

**DOI:** 10.1093/bioinformatics/btaf445

**Published:** 2025-08-09

**Authors:** Qinze Yu, Chang Zhou, Jiyue Jiang, Xiangyu Shi, Yu Li

**Affiliations:** Department of Computer Science and Engineering, CUHK, Hong Kong SAR 999077, China; Department of Computer Science and Engineering, CUHK, Hong Kong SAR 999077, China; Department of Computer Science and Engineering, CUHK, Hong Kong SAR 999077, China; Department of Computer Science and Technology, Beijing JiaoTong University, Beijing 100044, China; Department of Computer Science and Engineering, CUHK, Hong Kong SAR 999077, China; The CUHK Shenzhen Research Institute, Shenzhen 518057, China

## Abstract

**Motivation:**

Accurate and generalizable prediction of drug–target interactions (DTIs) remains a critical challenge for drug discovery, particularly when addressing underexplored targets and compounds. Recent advances in graph neural networks and large-scale pre-trained models offer new opportunities to capture rich structural and functional features essential for DTI prediction while enhancing the generalization ability.

**Results:**

We present GS-DTI, a graph structure-based DTI prediction framework that integrates molecular graph transformers, protein language models, and protein tertiary structure. Our method achieved robust and interpretable DTI predictions. GS-DTI extracts drug features from SMILES-derived molecular graphs using a knowledge-guided pre-trained transformer, while protein features are derived from both sequence and predicted 3D structure for comprehensive representation. A multi-task loss function equipped with contrastive learning is adopted to enhance generalization and functional interpretability. Extensive experiments on the benchmarks and challenging cross-domain settings demonstrate that GS-DTI achieves state-of-the-art performance. Notably, our model improves the MCC by over 10% compared to previous methods in the drug–target pair cold start test. The model can pinpoint the binding pockets of the targets, offering robust interpretability, and case studies show GS-DTI’s promising potential in virtual screening for new candidate drugs of BACE1.

**Availability and implementation:**

The GS-DTI source code and processed datasets are available at https://github.com/purvavideha/GSDTI. All experimental data are derived from public sources.

## 1 Introduction

Drug–target interaction (DTI) prediction is of great importance in the process of drug discovery (Chen *et al.* 2016, Huang *et al.* 2021b), as drugs exert their effects primarily by binding to specific protein targets. A drug is usually a chemical compound that induces physiological changes in the body when consumed, injected, or absorbed. A target, which is normally a protein, can be recognized or bound by substances such as ligands or drugs, enabling it to interact with or be influenced by these molecules. Traditional experimental methods for the measurement of drug–target interaction affinity are time- and cost-consuming, preventing analysis of data at scale (Deng *et al.* 2022). To improve efficiency, some in silico approaches were proposed. One class is docking simulations, which use the 3D structure of drugs and targets to identify their potential binding sites and binding affinity (Li *et al.* 2006, Pinzi and Rastelli 2019). However, these methods heavily rely on accurate 3D structure data, which may not be available for all samples, and they are based on well-defined binding sites, hence have difficulty detecting unknown sites. Second, there are similarity-based and network-based methods, which use protein-protein similarity, and drug–drug similarity to make inferences informed by the known DTI (Ding *et al.* 2014).

Similarity-based and network-based methods are not able to generalize to data that are absent from the training set. With the advancement of statistical and computational methodologies, machine learning frameworks were developed to resolve this problem. They achieved decent accuracy and enabled large-scale data processing in an acceptable time. The normal form of these methods is to extract the drug, target, and their interaction information and then systematically integrate them to build predictive models, which are dedicated to binary classification or regression tasks. For instance, DeepDTA (Öztürk *et al.* 2018), 3DProtDTA (Voitsitskyi *et al.* 2023), and DeepConv-DTI (Lee *et al.* 2019) extract molecular features of drugs and targets with Convolutional Neural Networks (CNN) and combine them for prediction. Considering the structures of drugs and targets are intrinsically graph-like, and the relationship between drugs and targets can be modeled with graphs, a wide range of Graph Neural Networks (GNN) based methods have been proposed. GraphDTA (Nguyen *et al.* 2021) and DrugBAN (Bai *et al.* 2023) take drug compounds as a graph of the interactions between atoms and utilize GNN for representation learning. ZeroBind (Wang *et al.* 2023) builds the graph of proteins based on the distance between amino acids in space. MINDG (Yang *et al.* 2024) constructs drug–target relationship graphs and uses Graph Attention Networks (GAT) for feature aggregation. Then, due to the improving availability of computational resources, molecule pre-trained models (Li *et al.* 2022) and protein language models (Elnaggar *et al.* 2022, Lin *et al.* 2023) are developed and show impressive performance on various downstream tasks (Yu *et al.* 2021, Shen *et al.* 2024). DLM-DTI (Lee *et al.* 2024) takes both molecule and protein pre-trained models for feature enrichment.

However, there are still some challenges with existing computational approaches for DTI prediction, which limit their effectiveness in real-world scenarios. First and foremost, they do not demonstrate robustness in domain-shift data prediction and may struggle to predict interactions involving rare drugs or targets that have low similarity to known data. The distribution of the training data can greatly vary from the distribution of the data in testing or actual applications. Therefore, the lack of generalizability can hinder the performance in identifying interactions for new or underexplored drug–target pairs. Furthermore, most methods lack interpretability, making it difficult to understand how the models make inferences. This black-box nature limits their utility in drug development, where understanding the molecular basis of drug–target interactions is crucial for designing effective therapies. Without clear explanations of DTI mechanisms, the model may not be considered trustworthy.

In this study, we addressed the above problems by leveraging the molecule graph and protein language models as well as the protein tertiary structures, and proposed a Graph Structure-based Drug Target Interaction prediction method (GS-DTI) for accurate DTI identification. Specifically, our model takes the Simplified Molecular Input Line Entry System (SMILES) of drugs and the protein amino acid sequences as input. For drugs, the SMILES strings are first converted into molecule graphs, and then a pre-trained molecular graph transformer (KPGT) (Li *et al.* 2022) is applied to represent embeddings of drug graphs. For targets, we used ESMFold (Lin *et al.* 2023) to predict the protein tertiary structures and obtain contact maps, and ESM-2 (Lin *et al.* 2023) to generate per-amino acid embeddings. We then built the protein graphs that use contact maps as edge information and amino acid embeddings as node features. After that, Graph Multiset Transformer (GMT) (Baek *et al.* 2021, Gu *et al.* 2023), a multi-head attention-based global pooling layer for capturing the interaction between nodes, is applied for protein message aggregation. The drug and target features are combined for DTI prediction. Here, we also adopted contrastive learning to enhance the representation quality and generalization of the drug and target.

We compared GS-DTI with several DTI prediction deep learning methods on both in-domain and cross-domain settings. The results demonstrated that our approach surpasses the other methods, achieving superior overall performance. Particularly, our model achieves more than 10% improvement of MCC over previous methods on the drug–target pair cold start test. Then, by checking the contribution of each amino acid for prediction, we found that GS-DTI could help detect the target binding pocket, providing interpretable insights for the prediction results. At last, we used GS-DTI to successfully identify effective inhibitors of BACE1, an unseen target to our model, indicating our model is a generalized and useful tool for discovering novel drugs for targets. These results suggest that GS-DTI can effectively predict DTI and greatly facilitate drug discovery.

## 2 Materials and methods

### 2.1 Datasets

#### 2.1.1 Training data

BindingDB (Liu *et al.* 2007) is a public, web-accessible database that provides information about experimentally measured binding affinities, primarily focusing on the interactions between proteins (commonly regarded as drug targets) and small, drug-like molecules. We access the data through the Therapeutics Data Commons (TDC) (Huang *et al.* 2021a). We used interactions in the BindingDB dataset that are measured in $K_{d}$ for training. The data labels (affinity values) are first converted into $pK_{d}$ (log scale), and then we set the threshold as 7 to binarize them. Considering our computational resource limitations preclude structure prediction for proteins with particularly long sequences, we removed all interaction data whose target lengths are longer than 1000, which is approximately the maximum length that we can handle. The processed dataset contains 33,238 negative samples and 8,846 positive samples, including 9,833 unique drugs and 1,235 unique proteins (Table 1, available as supplementary data at *Bioinformatics* online).

**Table 1.** Performance (ROC-AUC) on BIOSNAP unseen drug and protein split tests.^a^

Settings	DeepConv-DTI	GraphDTA	DrugBAN	DLM-DTI	MolTrans	DTI-LM	SP-DTI	GS-DTI
Unseen drugs	0.847 ± 0.009	0.792 ± 0.012	0.872 ± 0.005	0.859 ± 0.009	0.853 ± 0.011	0.874 ± 0.009	0.894 ± 0.009	**0.901** ± **0.010**
Unseen proteins	0.766 ± 0.022	0.650 ± 0.024	0.771 ± 0.024	0.828 ± 0.020	0.770 ± 0.029	0.866 ± 0.019	0.873 ± 0.019	**0.884** ± **0.021**

a Bold indicates the best performance.

#### 2.1.2 Test data

Two datasets, named Davis and BIOSNAP, are used for the independent tests. Data in the Davis set are derived from experimental binding affinity measurements between kinase inhibitors and kinases. The affinities are measured using the $K_{d}$ in nanomolar (nM). The Davis data is particularly focused on kinase inhibitors, making it specific and suitable to be the cross-domain data for testing DTI prediction models. We converted the affinity values into $pK_{d}$ and also set the threshold as 7 for binarization. We removed the duplicates and the same data pairs in the training data, resulting in 1,374 positives and 13,061 negatives. Our test set has 68 unique drugs and 366 unique targets.

The BIOSNAP dataset is collected from MolTrans (Huang *et al.* 2021b) test sets. We adopted their unseen drug set and unseen protein set for model evaluation. Dataset statistics are provided in Table 1, available as supplementary data at *Bioinformatics* online.

### 2.2 Model development

**Figure 1.**
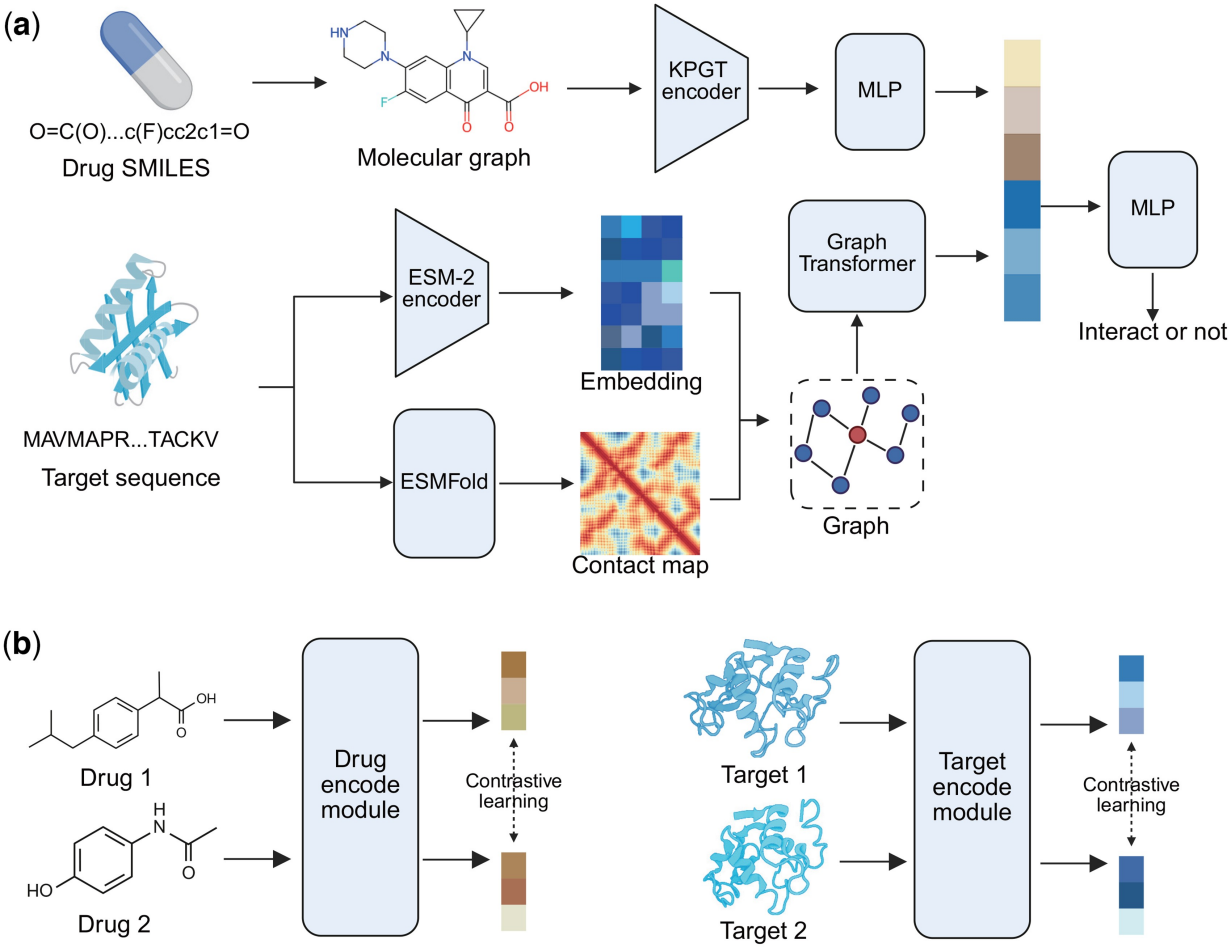
Framework of our proposed method GS-DTI. (a) The architecture of GS-DTI. Both drug and target are represented as graph structures in our model. We also applied pre-trained models to enhance the generalization ability of the model. (b) We designed an intra-contrastive learning between drugs and between targets to help GS-DTI learn generalizable representations.

We developed a DTI prediction model, which is named Graph Structure-based Drug–Target Interaction prediction method (GS-DTI). Our model takes SMILES of drugs and sequences of targets as input, and the architecture comprises two key components: the drug feature extraction module and the target feature extraction module, which are used to obtain the embeddings of our drugs and targets (Fig. 1a).

#### 2.2.1 Drug feature extraction module

The drug feature extraction module first gets molecular graphs of drug SMILES strings. For a graph G=(V,E), where $V={\left\{v_{i}\right\}}_{i\in [1,N_{v}]}$ is the set of nodes (atoms), $E={\left\{e_{i,j}\right\}}_{i,j\in [1,N_{v}]}$ is the set of edges (chemical bonds), and here $N_{v}$ is the number of nodes. Graphs are then fed into KPGT. KPGT proposed the Line Graph Transformer (LiGhT) structure, which focuses on chemical bonds and can capture the structural information of molecular graphs. The outputs of KPGT are 2304-dimensional embeddings of the input SMILES strings. The embeddings will be input to a two-layer Multilayer Perceptron (MLP) for information integration, and the finalized embeddings of drugs will be obtained.

#### 2.2.2 Target feature extraction module

We represented the protein as a graph G=(V,E), where *V* denotes the set of nodes and *E* represents the edges. To construct the graph, we first predicted the 3D structure of the target protein using ESMFold (Lin *et al.* 2023), an efficient protein structure prediction model without an MSA process. Based on the predicted 3D atomic coordinates, we generated a contact map to define the graph topology. Specifically, each residue in the protein is represented as a node in the graph, and an edge is created between two nodes if the distance between their $C_{\alpha }$ atoms is less than 8 Å. For the node, we extracted the per-residue embedding of the target from ESM-2 650M (Lin *et al.* 2023) as the feature for each node. The embeddings generated by ESM-2 contain rich evolutionary and functional information about proteins.

After protein graph construction, to extract meaningful representations from the protein graph, we employed a Graph Convolutional Network (GCN) with residual connections, followed by a Graph Multiset Transformer (GMT) (Baek *et al.* 2021) for graph-level embedding aggregation. The GCN captures local structural and relational information by iteratively updating node representations. Specifically, given a protein graph G=(V,E) with initial node features $H^{\left(0\right)}\in R^{|V|\times d}$, each GCN layer computes the node features as:


(1) $$H^{\left(k\right)}=\sigma \left(AH^{\left(k-1\right)}W^{\left(k\right)}\right)+H^{\left(k-1\right)},$$

where $A\in R^{\left|V|\times |V\right|}$ is the normalized adjacency matrix, $W^{\left(k\right)}\in R^{d\times d^{\prime }}$ is the learnable weight matrix of the *k*-th layer, σ(·) is the ReLU activation function, and the residual connection adds $H^{\left(k-1\right)}$ to the output of the current layer. This residual mechanism improves gradient flow and facilitates the learning of deeper networks. After *L* layers of GCN, where L=3 in our architecture, the final node embeddings $H^{\left(L\right)}$ are obtained.

To generate a fixed-size graph-level embedding from the variable-sized graph, we then applied a Graph Multiset Transformer (GMT). The GMT aggregates the node embeddings $H^{\left(L\right)}$ into a global representation $z_{G}$ while preserving important structural and relational information. Formally, the graph embedding is computed as:


(2) $$z_{G}=\text{GMT}\left(H^{\left(L\right)},A\right),$$

where GMT(·) denotes the multiset transformer mechanism, which uses attention to weight and aggregate node features dynamically. The resulting graph embedding $z_{G}$ serves as a compact and informative representation of the protein structure. The embeddings will also be input to a 2-layer MLP for information integration to generate the finalized protein embeddings.

At last, the drug embedding and target embedding were combined using a bilinear layer, and the resulting representation was used to predict DTI. The architecture hyperparameters of GS-DTI are listed in Table 2, available as supplementary data at *Bioinformatics* online.

**Table 2.** Ablation study of GS-DTI.^a^

	BACC	Precision	Recall	F1-score	MCC
GS-DTI w/o tertiary structure	0.756 ± 0.016	0.499 ± 0.013	0.598 ± 0.028	0.544 ± 0.022	0.494 ± 0.017
GS-DTI w/o GMT	0.771 ± 0.006	0.558 ± 0.013	0.592 ± 0.024	0.575 ± 0.017	0.529 ± 0.021
GS-DTI w/o KPGT	0.807 ± 0.009	0.538 ± 0.011	0.635 ± 0.018	0.565 ± 0.013	0.522 ± 0.019
GS-DTI w/o contrastive learning	0.792 ± 0.028	0.432 ± 0.046	0.668 ± 0.033	0.539 ± 0.035	0.492 ± 0.045
GS-DTI	**0.823** ± **0.014**	**0.615** ± **0.018**	**0.691** ± **0.015**	**0.651** ± **0.017**	**0.613** ± **0.015**

a Bold indicates the best performance.

#### 2.2.3 Loss function designed for the DTI prediction task

To address the challenges of data imbalance and the need for discriminative representation learning in DTI prediction, we proposed a novel loss function that integrates Focal loss (Lin *et al.* 2017) for the main DTI task and intra-contrastive learning (Chen *et al.* 2020) (Fig. 1b) as an auxiliary objective.

Given the inherent class imbalance in DTI datasets, we employed the focal loss to focus learning on hard-to-classify samples and mitigate the dominance of easy negatives. The focal loss is defined as:


(3) $$\begin{array}{c} L_{\text{focal}}=-\frac{1}{N}\sum_{i=1}^{N}[w{\left(1-{\hat{p}}_{i}\right)}^{t}y_{i}\;\text{log}({\hat{p}}_{i})\\ \;+\left(1-w){\hat{p}}_{i}^{t}(1-y_{i})\;\text{log}(1-{\hat{p}}_{i}\right)], \end{array}$$

where ${\hat{p}}_{i}=\sigma ({\hat{y}}_{i})$ is the predicted interaction probability from the sigmoid function, $y_{i}$ is the ground truth label, *w* balances positive and negative classes, and *t* adjusts the focus on hard examples.

To further enhance the discriminative power of the learned drug and target representations, we introduce an intra-contrastive learning loss based on a generalized NT-Xent loss. Unlike the original one, which defines positive pairs as two different augmentations of the same instance, our approach leverages domain-specific structural similarity to define positive pairs within a batch. Specifically, for a batch of *n* samples with normalized embeddings $z_{i}\in R^{d}$, we first compute the cosine similarity matrix among all embeddings. For each pair (i,j), a positive pair is defined if their structural similarity $S_{ij}$ (e.g., Tanimoto coefficient for drugs or TM-score for proteins) exceeds a given threshold θ. The loss for the batch is then computed as follows:


(4) $$\begin{array}{c} \text{sim}(z_{i},z_{j})=\frac{z_{i}\cdot z_{j}}{||z_{i}|{|}_{2}||z_{j}|{|}_{2}},\\ L_{ij}=\frac{\text{sim}(z_{i},z_{j})}{\tau },\\ M_{ij}=\{\begin{array}{cc} 1, & S_{ij}>\theta \;\text{and}\;i\neq j,\\ 0, & \text{otherwise}, \end{array} \end{array}$$

Here, $z_{i}$ and $z_{j}$ denote the normalized embeddings of samples *i* and *j*, respectively. τ is a temperature hyperparameter, Sij is the structural similarity between samples *i* and *j*, and θ is the threshold for defining positive pairs.

The generalized NT-Xent contrastive loss is:


(5) $$L_{\text{contrast}}=-\frac{1}{\sum_{i=1}^{n}\sum_{j=1}^{n}M_{ij}}\sum_{i=1}^{n}\sum_{j=1}^{n}M_{ij}\cdot \;\;\text{log}\;\frac{\;\text{exp}\;\left(L_{ij}\right)}{\sum_{k=1}^{n}\;\text{exp}\;\left(L_{ik}\right)},$$

where $M_{ij}$ indicates whether the pair (i,j) is considered a positive, and the denominator sums over all samples in the batch for normalization.

The final loss is:


(6) $$L_{\text{total}}=\alpha \cdot L_{\text{focal}}+\beta \cdot L_{\text{contrast}\_\text{drug}}+\gamma \cdot L_{\text{contrast}\_\text{target}},$$

where α, β, and γ are hyperparameters controlling the contribution of each component.

#### 2.2.4 Training details

For the training hyperparameters, we applied the Adam optimizer with the initial learning rate of 5$\times 10^{-5}$ and a weight decay of 1$\times 10^{-4}$. The total number of epochs is set to 50, with the early stopping strategy to get the best model during training. A batch size of 64 was used throughout the training. For the focal loss, the parameters were set to *w *= 0.5 and *t *= 2. For the intra-contrastive loss we applied, the structural similarity thresholds (θ) were set to 0.8 for drugs and 0.5 for proteins, with a temperature parameter τ = 0.07. The contributions of the focal loss and contrastive learning components were controlled by the hyperparameters α as 1, β and γ as 0.05.

For hyperparameter selection, we employed a grid search strategy by choosing the best-performing hyperparameter combination on 5-fold cross-validation on the training set. Specifically, we predefined a set of candidate values for each hyperparameter and systematically evaluated all possible combinations (Table 3, available as supplementary data at *Bioinformatics* online). All the experiments were run on four V100 GPU cards with 32GB of memory. For other included models, we used the hyperparameters they provided in the code repositories to train them. As GraphDTA (Nguyen *et al.* 2021) is originally trained for predicting the binding affinity, we changed its loss function to binary cross-entropy to make it a DTI prediction model.

**Table 3.** Prediction scores of 15 BACE1 inhibitors.

BACE1 inhibitor name	Prediction score
LY2811376	0.9992
Verubecestat	0.9761
LY2886721	0.9990
Epiberberine chloride	1.0000
Tasiamide B-11	0.0046
Lanabecestat	0.9994
Elenbecestat	1.0000
Atabecestat	0.9092
Scoulerine	0.9748
PF-06751979	0.9740
Tasiamide B-9	0.0392
AZD3839 free base	0.6613
LX2343	0.8584
BACE1-IN-5	0.9995
BACE1-IN-4	0.9984

### 2.3 Similarity estimation

For drug molecules, we applied Tanimoto Similarity to measure the structural similarity between drugs, which can be computed as:


(7) $$\mathrm{Tanimoto}(A,B)=\frac{A*B}{||A|{|}^{2}+||B|{|}^{2}-A*B},$$

where A and B are the feature vectors of drug A and drug B, respectively, and we adopted the Morgan fingerprint as the vector here.

To measure the structure similarity between proteins, we used the TM-scores calculated by TM-align (Zhang and Skolnick 2005). TM-score is independent of the protein size; it quantifies the similarity between two structures using a score ranging from (0, 1], where a score of 1 indicates a complete match between the structures.

To compute the sequence similarity between proteins, we adopted the Needleman–Wunsch algorithm provided by the “pairwise2” function from the Biopython library. This function optimizes the algorithm so that it can be better parallelized for higher efficiency.

## 3 Results

To evaluate the validity of our proposed method, we conducted the following experimental analyses. First, we performed 5-fold cross-validation to compare our approach with other DTI prediction methods. Next, to assess cross-domain generalizability, we evaluated the model on independent test sets using both overall and cold-start tests. Finally, we applied GS-DTI to identify binding sites for interpretability and to facilitate drug discovery for the BACE1 target.

### 3.1 GS-DTI generalizes to unseen drugs and targets

**Figure 2.**
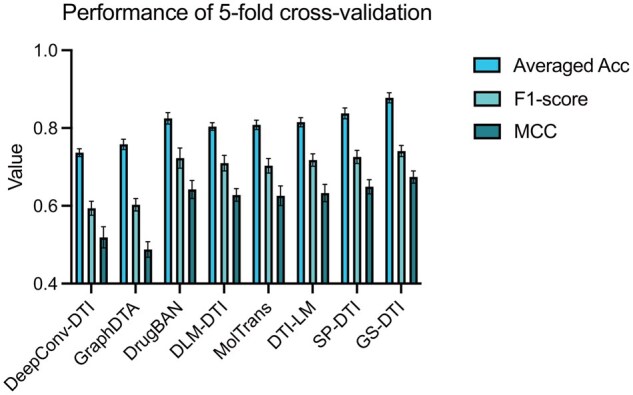
Performance of our model and included baseline methods on 5-fold cross-validation. Averaged Acc is the average value of positive and negative accuracy, indicating the model’s overall balanced classification ability across both classes.

We first trained and tested our model on the BindingDB (Liu *et al.* 2007) dataset, which contains 33,238 negative samples and 8,846 positive samples, including 9,833 unique drugs and 1,235 unique proteins. We compared GS-DTI with multiple baseline methods (Lee *et al.* 2019, Nguyen *et al.* 2021, Huang *et al.* 2021b, Bai *et al.* 2023, Lee *et al.* 2024, Ahmed *et al.* 2024, Liu *et al.* 2025) and calculated various evaluation metrics, including Balanced Accuracy, Precision, Recall, F1-score, and MCC. To achieve a fair comparison, all the models were retrained on the same data as ours. On the BindingDB dataset, all the models are tested with the stratified five-fold cross-validation strategy. Considering the negative data is obviously more than the positive data, we applied the stratified cross-validation to ensure each fold maintains the same proportion of positive and negative samples as the original dataset. From Fig. 2, all the models demonstrate stable performance across 5 folds. Our model has the best performance on Averaged Acc, F1-score, and MCC, demonstrating improved discriminative power in imbalanced contexts. Here, Averaged Acc is the averaged value of positive and negative accuracy.

Table 4, available as supplementary data at *Bioinformatics* online reports the average performance of methods on all 5 folds. Here, DeepConv-DTI has very high accuracy on negative data and achieves 0.966, but on positive data, the accuracy is merely 0.512. DeepConv-DTI did not take measures to address the data imbalance, making it not precise in predicting drug–target pairs that are with interactions. All other models except GraphDTA have accuracy on positive data above 0.7, and that of our model is even higher than 0.8, indicating these methods are more effective in handling data with DTI.

As cross-validation is within the in-domain data, all methods achieve decent performance. However, in the real-world application scenario, DTI prediction models are usually applied to drugs and targets that they have not seen in the training data. Therefore, generalization ability is essential for the model. To demonstrate the advantages of GS-DTI on unseen data, we first trained our model on the full BindingDB dataset, and then tested it on a domain-shift dataset, Davis (Davis *et al.* 2011), with multiple settings. The Davis data is particularly focused on kinase inhibitors, making it appropriate to simulate the model application.

**Figure 3.**
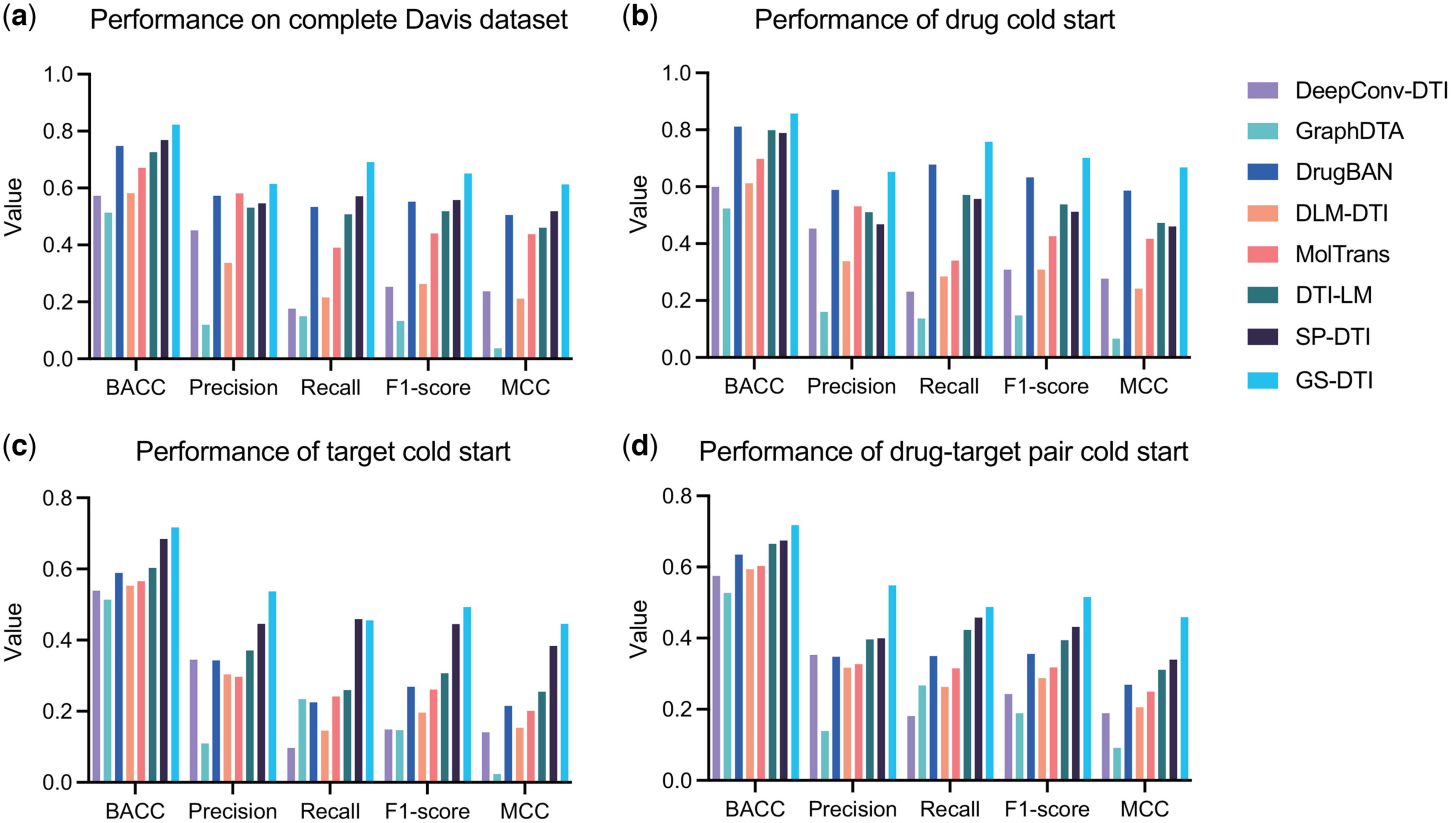
Performance comparison of GS-DTI and baseline methods on the Davis dataset under four evaluation settings: (a) full data, (b) drug cold-start, (c) target cold-start, and (d) drug–target pair cold-start.

Figure 3a and Table 5, available as supplementary data at *Bioinformatics* online, show the overall performance on the Davis dataset. Our model outperforms all baseline models across all BACC, Precision, Recall, F1-score, and MCC. Since the training data has a data imbalance problem, the negative data is 3 times more than the positive data. Some models are more inclined to learn from negatives. DeepConv-DTI, GraphDTA, and DLM-DTI have good performance on inactive drug–target pairs but are struggling to accurately predict data pairs with DTI, leading to low BACC and MCC. DrugBAN, MolTrans, DTI-LM, SP-DTI, and GS-DTI have a more balanced performance on both types, and our model is more precise and superior to the second-best model, SP-DTI, by about 0.05 on BACC.

Then, we applied three cold start settings, including drug cold start, target cold start, and drug–target pair cold start, on Davis data to comprehensively evaluate the models. For the drug cold start, all the drugs in the Davis dataset have no overlap with the training data. The results are presented in Fig. 3b and Table 6, available as supplementary data at *Bioinformatics* online. This time, GS-DTI still has the best performance across all metrics and achieves a BACC of 0.857. We substantially outperform all other methods by at least 0.08 on MCC. Although DLM-DTI, DTI-LM, and SP-DTI also learned drug information by taking molecule pre-trained models’ embeddings as input, GS-DTI has contrastive learning to further enhance the discriminative power of the learned drug representations. The cross-domain adaptation strategy enables DrugBAN to predict unseen drugs, making it the second-best model in the drug-cold start test.

In the scenario of the target cold start, we removed all the targets that existed in the training set. We found GS-DTI and SP-DTI outperform other models by a wider margin (Fig. 3c and Table 7, available as supplementary data at *Bioinformatics* online). The reason could be that both GS-DTI and SP-DTI model the structure of proteins and leverage embeddings generated by the protein language model, thus capturing richer structural and functional information than those that just take the pure amino acid sequence or pre-trained embeddings as input. In addition, our model has better BACC, Precision, F1-score, and MCC than SP-DTI, indicating robust performance in handling unseen targets.

For the drug–target pair cold start setting, none of the drugs and targets in this test overlapped with the training set, which allows the models to make inferences on completely unseen data. GS-DTI still wins the first place across all metrics and significantly outperforms other models for at least 0.120 on MCC (Fig. 3d and Table 8, available as supplementary data at *Bioinformatics* online).

**Figure 4.**
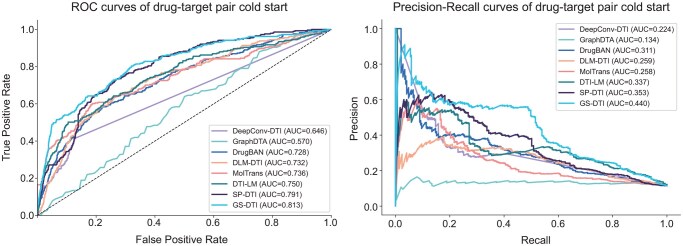
ROC curves and Precision-Recall curves of GS-DTI and baseline methods on the Davis drug–target pair cold-start test.

We also compared the AUROC (Area Under the Receiver Operating Characteristic Curve) and AUPRC (Area Under the Precision-Recall Curve) of all models under the drug–target pair cold start setting (Fig. 4). GS-DTI has comparable performance with the SOTA methods, demonstrating its ability to distinguish between interacting and non-interacting drug–target pairs. This consistent outperformance across multiple evaluation criteria highlights the robustness and generalizability of GS-DTI, suggesting that our approach is particularly effective in capturing the underlying patterns necessary for accurate drug–target interaction prediction.

**Figure 5.**
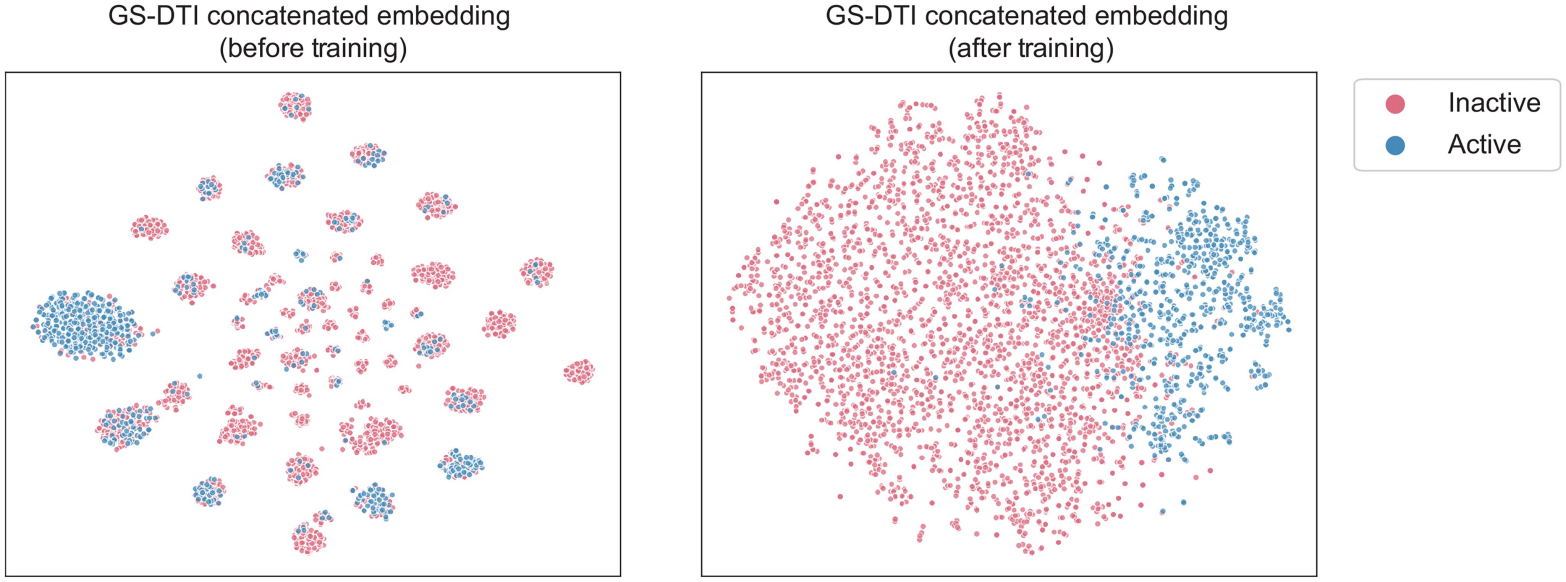
Visualization of concatenated drug–target pair embeddings using t-SNE before and after training. We took the Davis data as input and extracted the embeddings from the output of the bilinear layer.

Besides performance comparison, we then evaluated the representations learned by GS-DTI and visualized the concatenated embeddings of drug and target (Fig. 5) using t-distributed stochastic neighbor embedding (t-SNE) (Van der Maaten and Hinton 2008). Before training, the embeddings of active and inactive drug–target pairs are distributed in a scattered and largely overlapping manner. After training, the embeddings become much more structured, with a clear separation between the active and inactive samples. This indicates that the GS-DTI model learned informative representations, effectively mapping drug–target pairs with different activity labels into distinct regions of the embedding space.

Furthermore, we quantified the epistemic uncertainty of GS-DTI. Specifically, we applied Monte Carlo Dropout (Gal and Ghahramani 2016) and randomly sampled 100 pairs (50 positive and 50 negative) from each of four test settings on the Davis dataset. We performed 50 stochastic forward passes per sample to compute the variance of the model output as the uncertainty measure. This variance quantifies fluctuations in raw prediction scores, with higher values indicating greater model uncertainty. As shown in Fig. 1, available as supplementary data at *Bioinformatics* online, uncertainty was lowest for drug cold-start and modestly higher for target and pair cold-start compared to non-cold-start, with minimal gaps underscoring GS-DTI’s robustness in unseen data.

Subsequently, we assessed GS-DTI on the BIOSNAP dataset, which has a balanced data distribution, with drug- and target-cold start settings. Specifically, 20% of the drugs and targets, along with all DTI pairs involving them, were selected as the test set. Table 1 shows that GS-DTI has superior performance to SOTA deep learning baselines under both settings. The results are consistent with those in the Davis dataset, which suggests that GS-DTI can generalize to unseen drugs and proteins. For both drugs and targets, our model takes advantage of large-scale pre-trained models to boost the generalization ability and encode advanced features. Furthermore, considering the core role of structures in the drug–target interaction process, we adopted the structures for both the drug and target to make the model more precise. The combination of these techniques allows our model to handle unseen data well.

### 3.2 Ablation study to support the effectiveness of modules

In this section, we evaluated the contributions of different modules in GS-DTI by conducting an ablation study on the full Davis dataset. We proposed 4 ablation models. First, to examine the gain brought by incorporating protein structural information, we removed the ESMFold module. In this variant, target features were extracted solely from ESM-2, and an MLP was added to process the embeddings, which were then concatenated with the drug embeddings; other modules remained unchanged. We refer to this model as GS-DTI w/o tertiary structure. Second, we excluded the graph multiset transformer (GMT), utilizing only the GCN for molecular representation (GS-DTI w/o GMT), to assess the enhancement provided by the transformer module. Third, in GS-DTI w/o KPGT, we replaced the KPGT embeddings with Morgan fingerprints derived from SMILES, allowing us to evaluate the effectiveness of the pre-trained molecular graph encoder. The final ablation model, GS-DTI w/o contrastive learning, was trained without the contrastive learning objective to examine its impact on model performance.

The performance of all ablation models is summarized in Table 2. The results clearly demonstrate that protein structural information is essential for our model. Excluding it leads to a substantial performance drop. This decline is likely because ESM-2 embeddings are averaged across the sequences to obtain the same length features for all sequences, which merely contain sequence-level information. Nevertheless, the graph structure includes the residual-level protein information, implicitly promoting our model to learn the binding sites on targets.

Similarly, GS-DTI w/o GMT shows inferior performance, indicating that removing the graph transformer makes the model too shallow to effectively capture complex structural information.

Replacing the KPGT feature with the Morgan fingerprint also results in performance degradation, though not as pronounced. While the Morgan fingerprint does not leverage a pre-trained drug encoder, it still encapsulates the molecule’s two-dimensional topological information, which remains useful for this task.

The exclusion of contrastive learning (GS-DTI w/o contrastive learning) leads to a substantial decline in precision, suggesting that it plays a crucial role in enhancing the discriminative power of the learned representations, particularly in distinguishing positive from negative samples.

The above findings offer robust evidence supporting the design choices underlying GS-DTI and highlight the contribution of each module to the model’s performance.

### 3.3 GS-DTI detects binding pockets of targets

To demonstrate that our model is able to detect the binding pocket in the protein, we applied the grad-CAM (Selvaraju *et al.* 2017) on the output of the last graph convolutional layer to identify the important residues that make more contributions to the prediction. The PDBbind database (Wang *et al.* 2005) systematically integrates experimentally measured binding affinity data with three-dimensional structural information of biomolecular complexes from the Protein Data Bank (PDB) (Berman *et al.* 2000). We sampled 2 drug–target pairs in PDBbind (PDB IDs: 4HGE and 3L7B) and checked if the important amino acids are aligned with the pockets of targets. Specifically, we visualized the complex structures, the ground truth pocket, and the GS-DTI predicted important sites.

**Figure 6.**
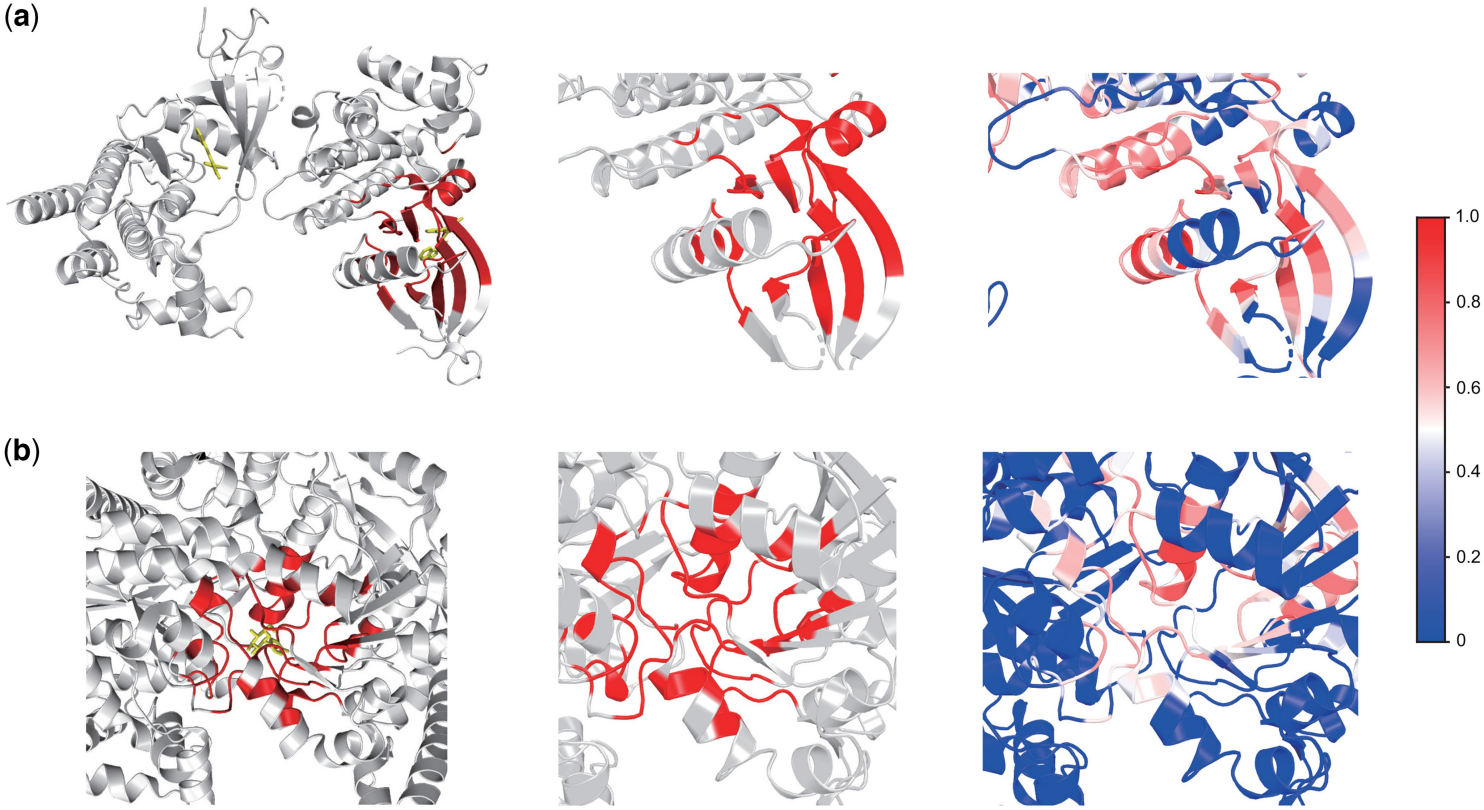
Visualization of the tertiary structure of protein–ligand complex and binding pockets. For each subfigure, the left is the protein–ligand complex structure. The middle is the binding pocket structure alone. The right is the binding pocket and surrounding regions. (a) Visualization for a complex with PDB ID 4HGE. (b) Visualization for a complex with PDB ID 3L7B.

4HGE (Fig. 6a), a crystal structure of the JAK2 tyrosine kinase (JH1 domain) in complex with the small molecule inhibitor compound 8, providing insights into selective kinase inhibition pertinent to cancer and inflammatory disease therapies (Hanan *et al.* 2012). GS-DTI’s high-weight regions closely correspond to the binding pocket regions in the 4HGE crystal structure (Fig. 2, available as supplementary data at *Bioinformatics* online). When the proportion of top-ranked residues selected matches the proportion of annotated binding sites in the protein, the recall—the fraction of true binding residues correctly identified—is 0.415, indicating the model’s ability to reveal the fine-grained drug–target interaction information.

3L7B (Fig. 6b), a crystal structure of glycogen phosphorylase (a key enzyme in glucose metabolism) in complex with the synthetic inhibitor DK3, illustrates the molecular basis for allosteric inhibition of this metabolic enzyme (Tsirkone *et al.* 2010). In the 3L7B structure, DK3 binds to a distinct allosteric site, with its aromatic and hydrophobic moieties fitting tightly into complementary pockets of the enzyme surface, stabilizing an inactive conformation. It could be observed that the residues that make a great contribution to the prediction are highly similar to the binding sites of glycogen phosphorylase (Fig. 3, available as supplementary data at *Bioinformatics* online). Under the same setting, the recall is 0.571, further demonstrating the model’s effectiveness in identifying true binding sites.

These alignments suggest that our model effectively captures critical interaction sites, highlighting its potential in implicitly predicting the binding pocket of targets.

### 3.4 GS-DTI facilitates drug discovery for BACE1

To further test the applicability of GS-DTI, we used it for Beta-site amyloid precursor protein cleaving enzyme 1 (BACE1) drug discovery by virtual screening. BACE1 is an aspartic-acid protease that plays a critical role in the production of β-amyloid peptides, which accumulate to form amyloid plaques—a hallmark of Alzheimer’s disease pathology. The inhibition of BACE1 can reduce β-amyloid generation and has therefore been extensively studied as a potential therapeutic strategy for Alzheimer’s disease and related neurodegenerative disorders (Cole and Vassar 2007). We collected the approved drugs in the DrugBank database (Knox *et al.* 2024) and removed the drugs from our training data for the virtual screening of potential BACE1 inhibitors, which resulted in 439 drugs. Notably, BACE1 is an unseen target since it is not in our training data. We also included 15 experimentally verified BACE1 inhibitors to evaluate the recall ability of GS-DTI, and there are a total of 454 molecules for screening.

The predicted results of the 15 inhibitors are listed in Table 3. We found that our model could successfully rediscover 13 out of the 15 (86.7%) inhibitors, and the remaining two are both derivatives of Tasiamide B, a peptide that serves as the Cathepsin D inhibitor, and not specific for binding with BACE1 (Li *et al.* 2019). It’s worth noting that 10 of the 15 BACE1 inhibitors were recalled in the top-60 potential drugs by GS-DTI. Additionally, our model predicted that 43/439 drugs in DrugBank have a high probability (confidence score >0.99, Table 9, available as supplementary data at *Bioinformatics* online) to bind to BACE1. We found that among these high-confidence candidates, doxercalciferol (Thiel *et al.* 2023), alfacalcidol (Thiel *et al.* 2023), and penbutolol (Pauls *et al.* 2021) had been verified to be the BACE1 inhibitors. Fosamprenavir is an HIV protease inhibitor, which is a class of drugs that are structurally related to BACE1 inhibitors (Mulato *et al.* 2024). These literatures provide evidence that GS-DTI is effective for BACE1 inhibitor virtual screening.

We also visualized the embeddings of 15 verified inhibitors and 439 drugs for screening (Fig. 4 at *Bioinformatics* online). From the results, 13 out of 15 GS-DTI identified known BACE1 inhibitors cluster closely with the high-confidence candidate drugs. The proximity of their distance indicates that our model effectively captures structural and functional features relevant to BACE1 inhibition. Furthermore, other candidates and predicted non-inhibitors form distinct clusters, suggesting that GS-DTI can robustly separate likely inhibitors from non-inhibitors based on the learned representations. The clustering supports that the embedding space encodes meaningful biochemical information relevant to BACE1 activity. The above findings demonstrate that GS-DTI can serve as a valuable tool to accelerate the drug development process.

## 4 Conclusion

In this study, we proposed GS-DTI, a framework that incorporates the structural information of both drugs and proteins for DTI prediction. Comprehensive experimental analysis indicates our proposed method achieved better performance on unseen drugs and targets. Furthermore, by identifying residues that make important contributions to the prediction, our model can provide interpretable insights for the binding pockets. GS-DTI also shows promising potential in discovering novel drug candidates and serves as a powerful tool for drug development.

Our study, like many DTI prediction works, relies on $K_{d}$ values for labels, which vary due to assay protocols. To mitigate this, we converted $K_{d}$ to $pK_{d}$ and binarized the labels, focusing on classification rather than regression. However, some assay-dependent variability may remain and could affect model performance and transferability. In addition, this binary classification may lose granularity by merging weak and non-binders, overlooking affinity nuances for tasks like lead optimization. Future work may consider addressing this by incorporating assay metadata or using more standardized benchmarking datasets. Besides, extending the framework to multi-class frameworks (e.g., categorizing into strong, weak, and non-binders) to preserve finer details from $pK_{d}$ data is worth investigation.

In the future, we plan to further enhance our model and develop a more comprehensive and automated pipeline for drug discovery. We will also focus on improving the scalability and efficiency of GS-DTI, enabling large-scale virtual screening and accelerating the identification of promising lead compounds.

## Supplementary Material

btaf445_Supplementary_Data
