# Supplementary material for: GS-DTI: a graph-structure-aware framework leveraging large language models for drug–target interaction prediction
Source: Bioinformatics. 2025 Aug 9;41(8):btaf445. doi: 10.1093/bioinformatics/btaf445 (PMC12396372; doi:10.1093/bioinformatics/btaf445)
Supplement: btaf445_Supplementary_Data [file btaf445_supplementary_data.pdf]

Supplementary Information for: GS-DTI: A Graph-Structure-Aware Framework Leveraging Large Language Models for Drug–Target Interaction Prediction

Qinze Yu*[1], Chang Zhou*[1], Jiyue Jiang[1], Xiangyu Shi[2], and Yu Li †[1,3]

[1]Department of Computer Science and Engineering, CUHK, Hong Kong SAR, China
[2]Department of Computer Science and Technology, Beijing JiaoTong University, Beijing, China
[3]The CUHK Shenzhen Research Institute, Hi-Tech Park, Nanshan, Shenzhen, 518057, China

List of Supplementary Notes

1 Dataset 2

2 Hyperparameters 2

3 Performance evaluation metrics 2

4 Model performance 3

5 Interpretability of GS-DTI 5

6 Virtual screening for drug discovery 7

*Equal first authorship.
†Corresponding Author. Email: liyu@cse.cuhk.edu.hk

1 Dataset

We took BindingDB as our training set and tested the model on the Davis dataset. It could be observed that there exists a data imbalance problem where negative data are times more than the positive data (Supplementary Table 1). We also included BIOSNAP as the second test set to evaluate our model.

Supplementary Table 1: Statistics of DTI datasets used in this study

Dataset	Number of data			
	Drug	Target	Positive pair	Negative pair
BindingDB	9,833	1,235	8,846	33,238
Davis	68	366	1,374	13,061
BIOSNAP unseen drug train	3,588	2,178	9,535	9,619
BIOSNAP unseen drug test	902	1,796	2,918	2,675
BIOSNAP unseen protein train	4,383	1,745	9,876	9,499
BIOSNAP unseen protein test	2,931	436	2,578	2,762

2 Hyperparameters

The key hyperparameters and architectural details of GS-DTI (pre-trained modules are excluded) are shown in Supplementary Table 2.

Supplementary Table 2: Architectural Hyperparameters of GS-DTI (excluding pretrained modules)

Module	Description	Input/Output Dim	Key Hyperparameters
**GraphCNN**	Protein graph encoder	Input: 512, Output: 512	3 GCNConv layers, ReLU, residual, dropout=0.2
	Pooling layer	512→256	GraphMultisetTransformer (8 heads, dropout=0.25)
**Drug MLP**	Drug feature encoder	Input: drug_dim, Output: 256	Linear(drug_dim, 1024) → ReLU → Dropout(0.2) → Linear(1024, 256)
**Bilinear Interaction**	Drug-protein fusion	Input: (256, 256), Output: 256	Bilinear(256, 256, 256)
**Output MLP**	Prediction head	Input: 256, Output: output_dim	Linear(256, output_dim)

For the training hyperparameters, we tuned them based on the grid search. And the search space is shown in Supplementary Table 3.

Supplementary Table 3: Grid Search Space for Hyperparameter Selection

**Hyperparameter**	**Symbol**	**Search Space**	**Best Value**
Learning Rate	η	$\{1 \times 10^{-4}, 5 \times 10^{-5}, 1 \times 10^{-5}\}$	5×10^{-5}
Weight Decay	λ	$\{1 \times 10^{-4}, 5 \times 10^{-5}, 1 \times 10^{-5}\}$	1×10^{-4}
Batch Size	B	$\{16, 32, 64\}$	64
Focal Loss Weight	w	$\{0.25, 0.5, 0.75\}$	0.5
Focal Loss Gamma	t	$\{1, 2, 3\}$	2
Drug Similarity Threshold	θ_{drug}	$\{0.5, 0.6, 0.7, 0.8, 0.9\}$	0.8
Protein Similarity Threshold	θ_{protein}	$\{0.4, 0.5, 0.6\}$	0.5
Temperature Parameter	τ	$\{0.05, 0.07\}$	0.07
Focal Loss Weight	α	$\{1\}$	1
Drug Contrastive Loss Weight	β	$\{0.01, 0.05, 0.1\}$	0.05
Target Contrastive Loss Weight	γ	$\{0.01, 0.05, 0.1\}$	0.05

3 Performance evaluation metrics

Since drug-target interaction prediction is a binary classification and has the problem of data imbalance, we take various evaluation metrics to achieve a fair and comprehensive performance comparison that could reflect the

prediction capacity of models on the minor class. Specifically, the following metrics are used:

$$\text{Accuracy} = \frac{TP + TN}{TP + FP + TN + FN}, \tag{1}$$

$$\text{Balanced Accuracy} = \frac{1}{2} \times \left(\frac{TP}{TP + FN} + \frac{TN}{TN + FP} \right), \tag{2}$$

$$\text{Precision} = \frac{TP}{TP + FP}, \tag{3}$$

$$\text{Recall} = \frac{TP}{TP + FN}, \tag{4}$$

$$\text{F1-score} = 2 \times \frac{\text{Precision} \times \text{Recall}}{\text{Precision} + \text{Recall}}, \tag{5}$$

$$\text{MCC} = \frac{TP \times TN - FP \times FN}{\sqrt{(TP + FP) \times (TP + FN) \times (TN + FP) \times (TN + FN)}}, \tag{6}$$

where TP denotes the number of true positives, TN denotes the number of true negatives, FP denotes the number of false positives, and FN denotes the number of false negatives. All used macro average values to eliminate the influence of data imbalance.

4 Model performance

We evaluated the models first with the 5-fold cross-validation on the BindingDB dataset (Supplementary Table 4). We tested the performance of our model and other included models on the complete Davis dataset (Supplementary Table 5), the Davis dataset with drug cold start setting (Supplementary Table 6), the Davis dataset with target cold start setting (Supplementary Table 7), and the Davis dataset with drug cold start setting (Supplementary Table 8). GS-DTI outperforms other methods in predicting unseen data and thus has good generalization ability.

Supplementary Table 4: The average performance of cross-validation experiment. 'Acc on pos' and 'Acc on neg' are the accuracy on positive and negative data.

	Acc on pos	Acc on neg	F1-score	MCC
DeepConv-DTI	0.512±0.008	**0.966**±0.013	0.599±0.018	0.517±0.027
GraphDTA	0.644±0.019	0.871±0.008	0.601±0.016	0.487±0.020
DrugBAN	0.767±0.013	0.882±0.017	0.722±0.026	0.640±0.023
DLM-DTI	0.726±0.006	0.882±0.015	0.710±0.020	0.628±0.016
MolTrans	0.745±0.012	0.871±0.013	0.703±0.019	0.626±0.025
DTI-LM	0.753±0.010	0.876±0.015	0.718±0.016	0.633±0.022
SP-DTI	0.785±0.011	0.890±0.018	0.726±0.017	0.649±0.018
GS-DTI	**0.861±0.014**	0.895±0.012	**0.741±0.014**	**0.674±0.016**

Supplementary Table 5: Performance comparison of different models on the Davis dataset.

	BACC	Precision	Recall	F1-score	MCC
DeepConv-DTI	0.573±0.013	0.451±0.019	0.176±0.025	0.253±0.025	0.237±0.029
GraphDTA	0.514±0.018	0.120±0.024	0.150±0.016	0.133±0.022	0.037±0.009
DrugBAN	0.748±0.005	0.573±0.009	0.534±0.015	0.552±0.010	0.505±0.018
DLM-DTI	0.582±0.023	0.337±0.028	0.215±0.035	0.263±0.024	0.211±0.029
MolTrans	0.671±0.021	0.581±0.020	0.390±0.028	0.440±0.028	0.438±0.025
DTI-LM	0.726±0.013	0.531±0.014	0.508±0.017	0.519±0.015	0.460±0.013
SP-DTI	0.769±0.015	0.546±0.019	0.571±0.026	0.558±0.025	0.519±0.024
GS-DTI	**0.823±0.014**	**0.615±0.018**	**0.691±0.015**	**0.651±0.017**	**0.613±0.015**

Supplementary Table 6: Performance comparison of different models on the Davis dataset with the drug cold start setting.

	BACC	Precision	Recall	F1-score	MCC
DeepConv-DTI	0.600±0.024	0.453±0.041	0.231±0.024	0.309±0.028	0.277±0.033
GraphDTA	0.524±0.018	0.160±0.032	0.137±0.027	0.148±0.030	0.066±0.021
DrugBAN	0.811±0.018	0.589±0.029	0.678±0.021	0.633±0.028	0.586±0.023
DLM-DTI	0.612±0.025	0.338±0.029	0.285±0.036	0.309±0.035	0.242±0.027
MolTrans	0.698±0.022	0.531±0.033	0.340±0.026	0.426±0.030	0.417±0.022
DTI-LM	0.799±0.023	0.510±0.019	0.571±0.023	0.538±0.016	0.473±0.024
SP-DTI	0.789±0.016	0.468±0.023	0.557±0.027	0.512±0.024	0.460±0.029
GS-DTI	**0.857±0.020**	**0.652±0.021**	**0.758±0.016**	**0.701±0.019**	**0.668±0.023**

Supplementary Table 7: Performance comparison of different models on the Davis dataset with the target cold start setting.

	BACC	Precision	Recall	F1-score	MCC
DeepConv-DTI	0.539±0.030	0.345±0.039	0.097±0.021	0.149±0.033	0.141±0.024
GraphDTA	0.514±0.021	0.110±0.017	0.234±0.025	0.147±0.020	0.024±0.015
DrugBAN	0.589±0.008	0.343±0.014	0.225±0.013	0.269±0.014	0.215±0.011
DLM-DTI	0.553±0.017	0.304±0.023	0.146±0.015	0.196±0.021	0.154±0.019
MolTrans	0.566±0.015	0.297±0.026	0.242±0.020	0.251±0.014	0.201±0.017
DTI-LM	0.603±0.019	0.371±0.027	0.260±0.023	0.307±0.026	0.255±0.020
SP-DTI	0.684±0.023	0.446±0.024	**0.459±0.021**	0.445±0.026	0.384±0.028
GS-DTI	**0.717±0.023**	**0.537±0.026**	**0.456±0.027**	**0.493±0.023**	**0.446±0.022**

After model evaluation, we also quantified the epistemic uncertainty of GS-DTI using Monte Carlo Dropout. We randomly sampled 100 samples (50 positive and 50 negative data pairs) for each of the 4 test settings (not cold-start, drug cold-start, target cold-start, and drug-target pair cold-start) on the Davis dataset. For each sample, we performed 50 stochastic forward passes with dropout enabled, directly computing the variance of the model output (logits) as the uncertainty value. This variance measures the fluctuation in the model's raw prediction scores, with higher values indicating greater epistemic uncertainty. Supplementary Figure 1 illustrates the uncertainty distributions across cold-start categories. Data under drug cold-start exhibit the lowest uncertainty (mean:0.0846). Target cold-start (mean:0.1692), and drug-target pair cold-start (mean:0.2005) showed higher uncertainty than the non-cold-start setting (mean:0.1677). However, the gap is not significant, indicating the robustness of GS-DTI when predicting unseen data.

Supplementary Table 8: Performance comparison of different models on the Davis dataset with the drug-target pair cold start setting.

	BACC	Precision	Recall	F1-score	MCC
DeepConv-DTI	0.575±0.021	0.353±0.026	0.181±0.042	0.243±0.037	0.189±0.034
GraphDTA	0.527±0.035	0.139±0.046	0.267±0.033	0.189±0.044	0.092±0.039
DrugBAN	0.635±0.028	0.348±0.031	0.350±0.041	0.356±0.035	0.269±0.038
DLM-DTI	0.594±0.026	0.317±0.044	0.263±0.028	0.288±0.035	0.206±0.027
MolTrans	0.603±0.018	0.327±0.036	0.315±0.038	0.318±0.032	0.250±0.028
DTI-LM	0.665±0.017	0.396±0.019	0.423±0.025	0.394±0.021	0.311±0.015
SP-DTI	0.674±0.023	0.399±0.028	0.457±0.026	0.431±0.025	0.339±0.023
GS-DTI	**0.718±0.015**	**0.548±0.029**	**0.488±0.028**	**0.516±0.025**	**0.459±0.027**

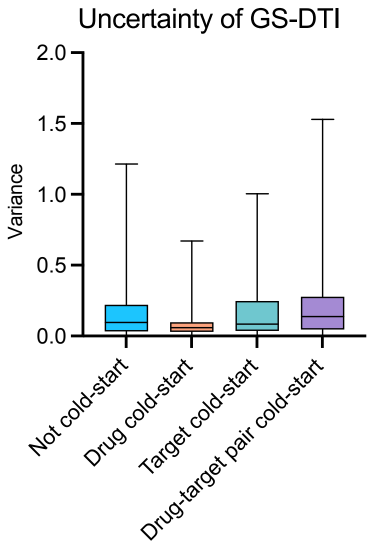

Supplementary Figure 1: Epistemic uncertainty estimation of GS-DTI. We computed the variance of model prediction using Monte Carlo Dropout under 4 test settings on the Davis dataset. Unpaired t-tests showed no significant differences between non-cold-start and target cold-start or drug-target pair cold-start conditions ($p > 0.05$).

5 Interpretability of GS-DTI

The prediction contribution weights for each amino acid of 4HGE and 3L7B are shown in Supplementary Figure 2 and 3. The binding pockets are well aligned with the amino acids that have high weights, indicating our model could help to detect the binding sites of targets.

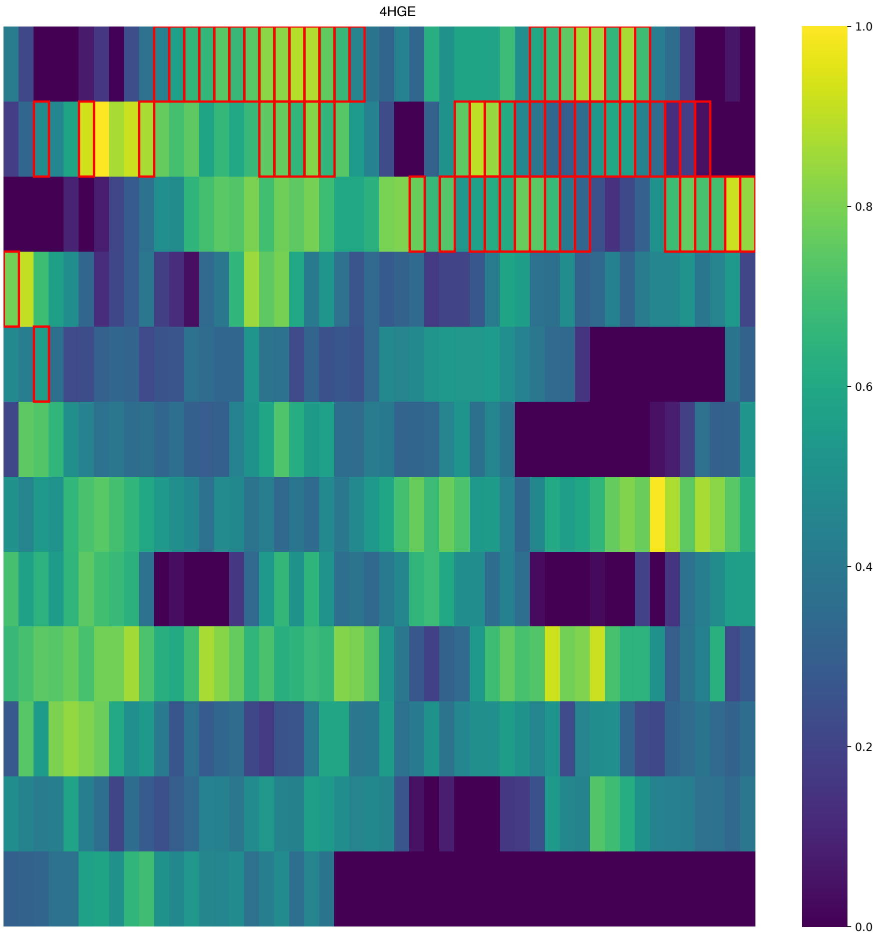

Supplementary Figure 2: Grad-CAM's prediction contribution weights of the target in the drug-target complex (PDB ID: 4HGE). Red boxes indicate binding site residues.

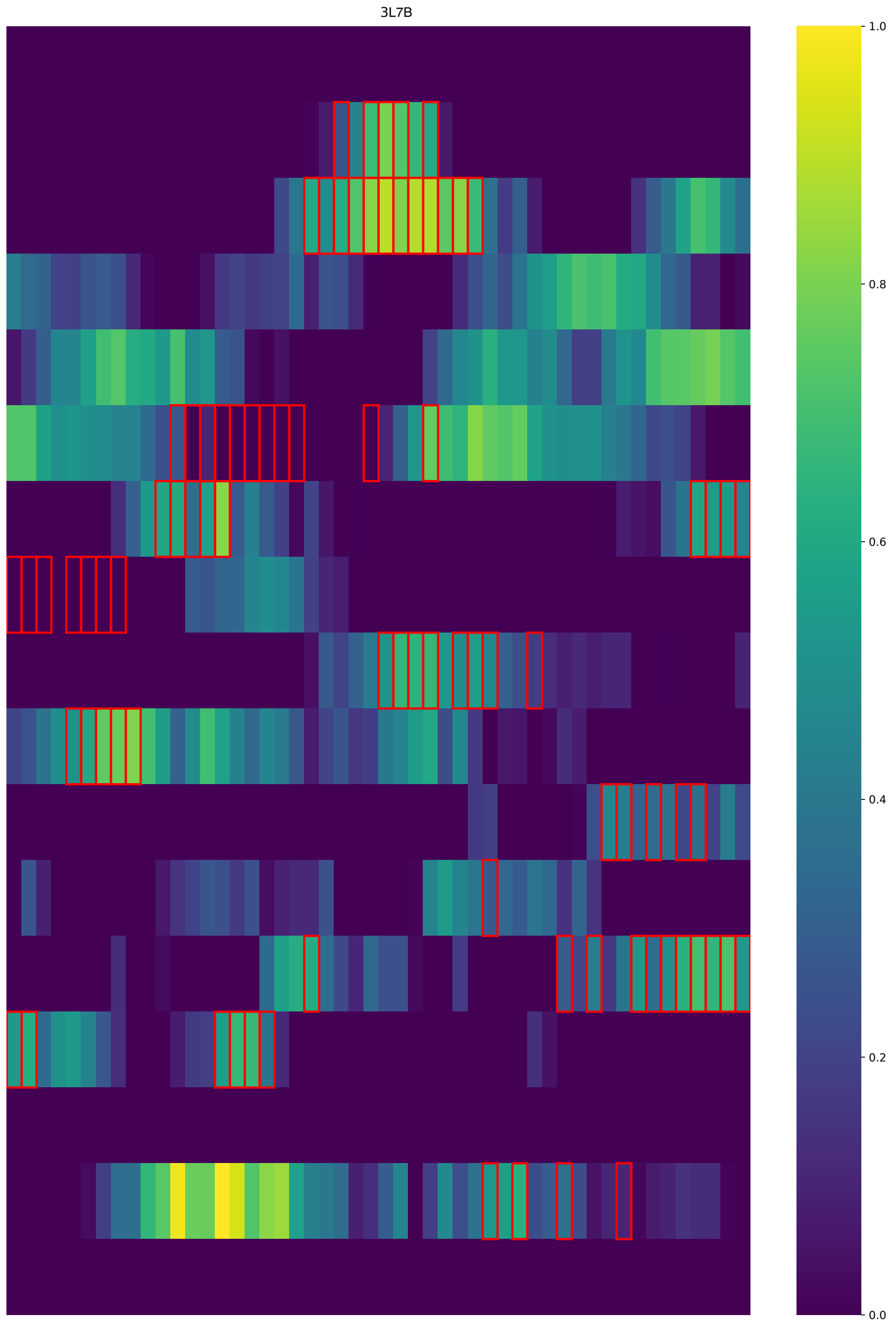

Supplementary Figure 3: Grad-CAM's prediction contribution weights of the target in the drug-target complex (PDB ID: 3L7B). Red boxes indicate binding site residues.

6 Virtual screening for drug discovery

GS-DTI predicted 43 out of 439 drugs in DrugBank have a high probability (prediction score > 0.99, Supplementary Table 9) to bind to BACE1. The higher prediction score here means the model has higher confidence in deciding that the drug can bind to BACE1. Considering the high recall rate of our method, these 43 drugs have great potential to be the BACE1 inhibitors and deserve further investigations.

From Supplementary Figure 4, 13 of 15 known BACE1 inhibitors (orange) cluster closely with the high-confidence candidate drugs (blue). Furthermore, other candidates (pink) and predicted non-inhibitor (green) form distinct clusters, suggesting that the model can robustly separate likely inhibitors from non-inhibitors based on the

learned representations.

Supplementary Table 9: The 43 predicted BACE1 inhibitors from DrugBank with a confidence score of more than 0.99.

Name	Prediction_score
Histrelin	0.99999994
Liotrix	0.99999934
Buserelin	0.999999
Norgestrel	0.99999726
Homatropine methylbromide	0.9999966
Fosamprenavir	0.99999213
Clobetasone	0.9999898
Methscopolamine bromide	0.99998915
Goserelin	0.999982
Tibolone	0.99997973
Ivermectin	0.9999658
Ioflupane I-123	0.9999655
Rolapitant	0.9999301
Oxymetholone	0.9998874
Metipranolol	0.99988395
Paritaprevir	0.9998564
Phenylbutazone	0.9997907
Doxercalciferol	0.99977183
Glecaprevir	0.9997311
Anileridine	0.99971825
Oxandrolone	0.9996845
Alfacalcidol	0.999662
Clocortolone	0.9996567
Estradiol cypionate	0.99955153
Permethrin	0.9993525
Tolvaptan	0.99931437
Chloramphenicol palmitate	0.99921554
Penbutolol	0.9986265
Norelgestromin	0.9985031
Velpatasvir	0.9982782
Fosaprepitant	0.9981234
Triptorelin	0.99809325
Testosterone cypionate	0.9971679
Quazepam	0.99609584
Fidaxomicin	0.99600846
Anisotropine methylbromide	0.99594575
Trospium	0.99592733
Iodipamide	0.994854
Spinosad	0.9939856
Ledipasvir	0.9932072
Prazepam	0.99191535
Vinorelbine	0.9916088
Finafloxacin	0.9912536

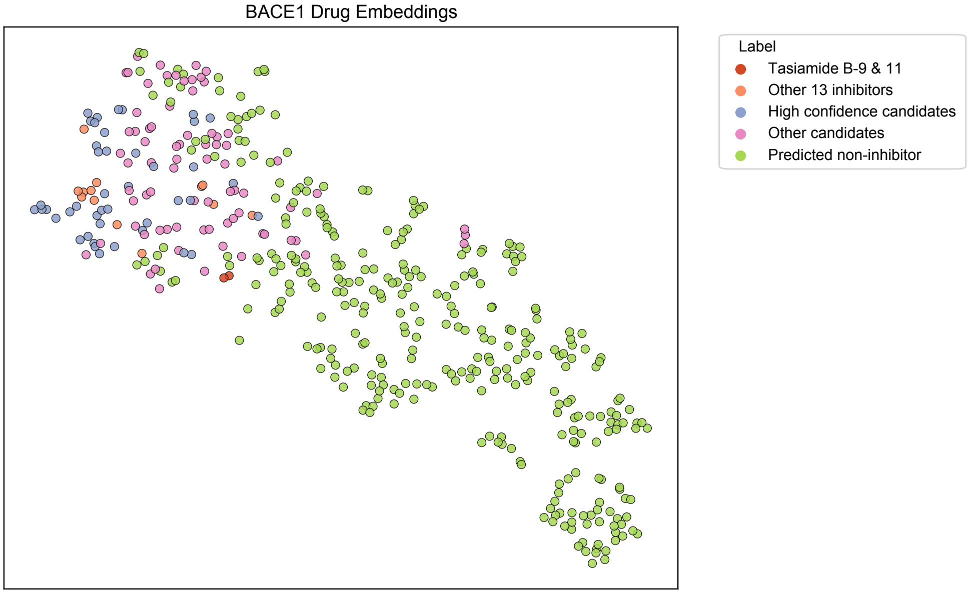

Supplementary Figure 4: Visualization of the embeddings of 15 validated inhibitors and 439 drugs for screening using t-SNE. We divided them into 5 groups: 1. Two Tasiamide B inhibitors that GS-DTI did not identify; 2. Thirteen inhibitors GS-DTI correctly predicted; 3. Forty-three high-confidence inhibitor candidates with predicted score higher than 0.99; 4. Other predicted inhibitors; 5. The drugs predicted as non-inhibitors.
